#  Screening for Excessive Alcohol Use and Brief Counseling of Adults — 17 States and the District of Columbia, 2014

**DOI:** 10.15585/mmwr.mm6612a1

**Published:** 2017-03-31

**Authors:** Lela R. McKnight-Eily, Catherine A. Okoro, Roberto Mejia, Clark H. Denny, John Higgins-Biddle, Dan Hungerford, Dafna Kanny, Joseph E Sniezek

**Affiliations:** ^1^Division of Congenital and Developmental Disorders, National Center on Birth Defects and Developmental Disabilities; ^2^Division of Population Health, National Center for Chronic Disease Prevention and Health Promotion, CDC.

Excessive and/or risky alcohol use* resulted in $249 billion in economic costs in 2010 (*1*) and >88,000 deaths in the United States every year from 2006 to 2010 (*2*). It is associated with birth defects and disabilities (e.g., fetal alcohol spectrum disorders [FASDs]), increases in chronic diseases (e.g., heart disease and breast cancer), and injuries and violence (e.g., motor vehicle crashes, suicide, and homicide).^†^ Since 2004, the U.S. Preventive Services Task Force (USPSTF) has recommended alcohol misuse screening and brief counseling (also known as alcohol screening and brief intervention or ASBI) for adults aged ≥18 years (*3*).^§^ Among adults, ASBI reduces episodes of binge-level consumption, reduces weekly alcohol consumption, and increases compliance with recommended drinking limits in those who have an intervention in comparison to those who do not (*3*). A recent study suggested that health care providers rarely talk with patients about alcohol use (*4*). To estimate the prevalence of U.S. adults who reported receiving elements of ASBI, CDC analyzed 2014 Behavioral Risk Factor Surveillance System (BRFSS) data from 17 states^¶^ and the District of Columbia (DC). Weighted crude and age-standardized overall and state-level prevalence estimates were calculated by selected drinking patterns and demographic characteristics. Overall, 77.7% of adults (age-standardized estimate) reported being asked about alcohol use by a health professional in person or on a form during a checkup, but only 32.9% reported being asked about binge-level alcohol consumption (*3*). Among binge drinkers, only 37.2% reported being asked about alcohol use and advised about the harms of drinking too much, and only 18.1% reported being asked about alcohol use and advised to reduce or quit drinking. Widespread implementation of ASBI and other evidence-based interventions could help reduce excessive alcohol use in adults and related harms.

BRFSS is an ongoing state-based, random-digit–dialed telephone survey of the noninstitutionalized U.S. adult population aged ≥18 years. Information is collected on a variety of health conditions, health practices, and risk behaviors, including alcohol use. CDC analyzed 2014 data from 17 states and DC that administered an optional five-question ASBI module.** All respondents were asked three alcohol use screening–related questions: 1) “You told me earlier that your last routine checkup was [within the past year/within the past 2 years]. At that checkup, were you asked in person or on a form if you drink alcohol?”; 2) “Did the healthcare provider ask you in person or on a form how much you drink?”; 3) “Did the healthcare provider specifically ask whether you drank [5 for men/4 for women] or more alcoholic drinks on an occasion?” All respondents were also asked, “Were you offered advice about what level of drinking is harmful or risky for your health?” Finally, persons who responded affirmatively to any of the first three aforementioned questions were asked, “Healthcare providers may also advise patients to drink less for various reasons. At your last routine checkup, were you advised to reduce or quit your drinking?” Binge drinkers were identified by their response to the question, “Considering all types of alcoholic beverages, how many times during the past 30 days did you have [5 for men/4 for women] or more drinks on an occasion?” Analyses were conducted to account for the complex sampling design. Weighted crude and age-standardized overall and state-level prevalence estimates were calculated by selected drinking patterns and demographic characteristics. Only age-standardized estimates are reported in the results section of this report. Wald chi square tests were used to determine significant within-group differences. Only significant differences are reported. The median cooperation rate for the 18 sites was 65.8%^††^ and median response rate was 42.7%.^§§^

Overall, 77.7% of persons reported being asked about alcohol use in person or by form, 68.8% reported being asked how much they drink, and 32.9% reported being asked about binge drinking (Table 1). The prevalence of being asked about binge drinking was higher among males (35.0%), persons with less than a high school diploma (40.1%), and binge drinkers (36.8%) in comparison to their counterparts. Non-Hispanic whites and Asian/Pacific Islanders were asked about binge drinking less than were non-Hispanic blacks, Hispanics, and American Indian/Alaskan Natives.

**TABLE 1:** Weighted crude and age-standardized* prevalence of U.S. adults who reported being asked an alcohol use screening–related question by a health care provider at last routine checkup in the past 2 years — Behavioral Risk Factor Surveillance System, 17 states and the District of Columbia,^†^ 2014

Characteristic	Asked about alcohol use (affirmative to question 1)			Asked how much alcohol (affirmative to question 2)			Asked about binge drinking (affirmative to question 3)		
	Sample size	Crude % (95% CI)	Age-standardized % (95% CI)	Sample size	Crude % (95% CI)	Age-standardized % (95% CI)	Sample size	Crude % (95% CI)	Age-standardized % (95% CI)
**Total**	**97,063**	**76.6 (75.9–77.3)**	**77.7 (76.9–78.5)**	**97,589**	**67.6 (66.8–68.4)**	**68.8 (67.9–69.6)**	**87,457**	**32.1 (31.2–32.9)**	**32.9 (31.9–33.9)**
**Sex**									
Male	39, 170	76.4 (75.3–77.6)	77.3 (76.1–78.5)	39,224	68.0 (66.8–69.3)	68.7 (67.3–70.1)	35,230	34.2 (32.8–35.6)	35.0 (33.5–36.6)
Female	57,893	76.8 (75.9–77.7)	78.1 (77.1–79.1)	58,365	67.2 (66.2–68.3)	68.9 (67.7–70.0)	52,227	30.3 (29.2–31.4)	31.2 (29.9–32.4)
**Age group (yrs)**									
18–24	4,350	76.1 (72.7–79.3)	—	4,307	58.9 (55.0–62.6)	—	4,013	24.8 (21.8–28.1)	—
25–34	7,385	84.2 (82.0–86.1)	—	7,265	76.0 (73.4–78.5)	—	6,236	37.5 (34.4–40.7)	—
35–44	10,326	82.3 (80.4–84.0)	—	10,184	75.4 (73.2–77.3)	—	8,536	37.4 (35.0–39.8)	—
45–64	38,930	78.9 (77.9–79.8)	—	38,859	72.0 (70.9–73.0)	—	34,016	34.6 (33.4–35.9)	—
≥65	36,072	64.0 (62.9–65.2)	—	36,974	54.3 (53.1–55.4)	—	34,656	25.2 (24.1–26.3)	—
**Race/ethnicity**									
White, non-Hispanic	74,533	77.0 (76.2–77.7)	79.2 (78.3–80.1)	75,099	68.8 (67.9–69.6)	71.3 (70.2–72.3)	66,377	29.4 (28.5–30.3)	31.2 (30.0–32.4)
Black, non-Hispanic	5,646	76.9 (74.3–79.3)	76.7 (74.1–79.1)	5,622	65.9 (62.7–68.9)	65.5 (62.2–68.6)	5,287	40.3 (37.1–43.6)	39.4 (36.2–42.7)
Hispanic	7,859	79.4 (77.1–81.5)	78.7 (76.5–80.8)	7,830	68.3 (65.7–70.8)	67.5 (65.1–69.9)	7,384	38.6 (35.8–41.4)	38.5 (35.9–41.3)
Asian/Pacific Islander	2,972	61.4 (56.5–66.1)	60.1 (55.6–64.4)	2,986	51.6 (46.6–56.6)	50.4 (46.1–54.7)	2,852	26.9 (22.3–32.0)	26.3 (22.0–31.2)
American Indian/Alaskan Native	1,788	79.0 (72.4–84.3)	79.3 (73.0–84.4)	1,799	70.2 (62.9–76.6)	68.6 (61.1–75.1)	1,695	39.8 (32.9–47.1)	39.4 (32.7–46.5)
Other non-Hispanic race or multiracial	2,873	76.7 (70.8–81.8)	76.0 (71.0–80.4)	2,862	67.7 (61.1–73.7)	68.9 (63.2–74.1)	2,607	34.6 (28.8–40.8)	37.2 (32.1–42.6)
**Education level**									
Less than high school diploma	6,793	72.7 (69.9–75.2)	74.0 (71.2–76.6)	6,790	62.5 (59.5–65.3)	64.0 (60.9–67.0)	6,569	39.6 (36.6–42.6)	40.1 (36.9–43.4)
High school diploma	25,748	72.9 (71.4–74.3)	74.9 (73.3–76.5)	26,048	61.9 (60.3–63.6)	64.6 (62.8–66.4)	24,250	31.7 (30.1–33.4)	33.4 (31.5–35.4)
College or tech school	64,200	79.3 (78.5–80.1)	80.0 (79.1–80.8)	64,425	71.5 (70.6–72.4)	72.1 (71.0–73.1)	56,334	30.4 (29.4–31.4)	31.0 (29.8–32.2)
**Disability status** ^§^									
Yes	28,117	74.8 (73.4–76.2)	77.7 (75.5–79.7)	28,450	67.3 (65.8–68.7)	71.2 (69.0–73.4)	26,240	33.4 (31.8–35.0)	35.0 (32.3–37.7)
No	68,208	77.3 (76.4–78.1)	77.7 (76.9–78.6)	68,402	67.8 (66.8–68.7)	68.4 (67.4–69.4)	60,566	31.6 (30.6–32.7)	32.3 (31.2–33.4)
**Insurance coverage**									
Yes	91,808	76.6 (75.8–77.3)	78.0 (77.2–78.8)	92,362	67.8 (67.0–68.6)	69.3 (68.3–70.2)	82,623	31.7 (30.8–32.6)	32.7 (31.6–33.7)
No	5,004	77.7 (74.8–80.3)	74.7 (71.8–77.5)	4,973	66.4 (63.1–69.5)	64.2 (61.0–67.3)	4,588	35.6 (32.3–39.0)	35.9 (32.8–39.2)
**Current drinker**									
Yes	50,422	81.3 (80.4–82.2)	81.6 (80.6–82.6)	50,492	74.4 (73.3–75.4)	74.3 (73.2–75.5)	43,641	32.3 (31.1–33.5)	32.3 (31.1–33.6)
No	45,417	71.4 (70.3–72.5)	73.5 (72.3–74.7)	45,859	60.1 (58.9–61.4)	62.8 (61.4–64.2)	42,716	31.8 (30.5–33.1)	33.6 (32.1–35.1)
**Binge drinker** ^¶^									
Yes	11,365	84.7 (83.0–86.3)	83.9 (82.3–85.4)	11,248	76.2 (74.0–78.2)	76.6 (74.7–78.4)	9,787	35.7 (33.4–38.1)	36.8 (34.6–39.0)
No	83,866	75.4 (74.6–76.2)	76.9 (76.0–77.7)	84,496	66.3 (65.4–67.1)	67.8 (66.8–68.8)	76,034	31.5 (30.5–32.4)	32.7 (31.6–33.8)

**Abbreviation:** CI = confidence interval.

* Estimates are age-standardized to the 2000 projected population for the United States.

^†^ Respondents were from 17 states (Connecticut, Florida, Hawaii, Indiana, Kansas, Kentucky, Massachusetts, Michigan, Minnesota, Montana, Nebraska, New Mexico, New York, Oregon, Texas, Washington, and Wisconsin) and the District of Columbia.

^§^ Respondents were asked, “Are you limited in any way in any activities because of physical, mental, or emotional problems?” and “Do you now have any health problem that requires you to use special equipment, such as a cane, a wheelchair, a special bed, or a special telephone?” Persons who responded yes to either question were classified as having a disability.

^¶^ Binge drinkers were defined as respondents who consumed ≥4 drinks per occasion during the preceding 30 days for women and ≥5 drinks for men. An occasion is generally defined as 2–3 hours.

Among binge drinkers, 37.2% reported being asked at least one of the alcohol use screening–related questions and advised about levels of drinking harmful or risky to their health (Table 2); prevalence was higher among males (43.8%) than females (27.6%) and among binge drinkers with disabilities (46.9%) than among those without disabilities (35.5%). Only 18.1% of binge drinkers who were asked at least one of the alcohol use screening–related questions were advised to reduce their drinking; in this group estimates were higher among males (22.6%) than females (11.4%), among American Indian/Alaska Natives (33.0%) than non-Hispanic whites (15.9%), among persons with a disability (30.1%) than among those without a disability (15.7%), and among persons with less than a high school education (31.3%) than among persons with a college or technical school education (14.1%). By state, the prevalence of binge drinkers being asked at least one of the alcohol use screening–related questions and being advised to reduce drinking ranged from 12.0% in Minnesota to 31.0% in DC (Table 3).

**TABLE 2:** Weighted crude and age-standardized* prevalence estimates of adult binge drinkers**^†^** who reported being asked an alcohol use screening–related question and advised about what level of drinking is harmful or risky for their health/advised to reduce their level of drinking by a health care provider at last routine checkup in the past 2 years — Behavioral Risk Factor Surveillance System, 17 states and District of Columbia,**^§^** 2014

Characteristic	Binge drinkers asked an alcohol use screening–related question					
	Advised on level of drinking harmful or risky to health			Advised to reduce drinking		
	Sample size	Crude % (95% CI)	Age-standardized % (95% CI)	Sample size	Crude % (95% CI)	Age-standardized % (95% CI)
**Total**	**9,620**	**36.4 (33.8–39.0)**	**37.2 (34.9–39.6)**	**9,855**	**17.3 (15.2–19.7)**	**18.1 (16.1–20.2)**
**Sex**						
Male	5,436	43.5 (39.8–47.2)	43.8 (40.5–47.1)	5,572	22.6 (19.4–26.1)	22.6 (19.8–25.7)
Female	4,184	25.8 (22.9–29.0)	27.6 (24.6–30.7)	4,283	9.6 (7.8–11.7)	11.4 (9.1–14.2)
**Age group (yrs)**						
18–24	963	38.7 (30.8–47.3)	—	988	15.3 (8.9–25.0) ^¶^	—
25–34	1,624	32.7 (27.4–38.5)	—	1,657	13.1 (9.5–17.8)	—
35–44	1,640	31.6 (27.0–36.5)	—	1,700	16.6 (12.8–21.3)	—
45–64	4,041	38.0 (34.5–41.7)	—	4,130	20.9 (17.7–24.5)	—
≥65	1,352	46.8 (41.2–52.5)	—	1,380	22.4 (17.8–27.9)	—
**Race/ethnicity**						
White, non-Hispanic	7,497	36.2 (33.3–39.2)	36.6 (34.0–39.3)	7,701	15.6 (13.1–18.5)	15.9 (13.7–18.5)
Black, non-Hispanic	421	37.8 (28.4–48.2)	39.6 (30.5–49.4)	424	23.0 (16.1–31.8)	25.2 (17.7–34.6)
Hispanic	838	35.0 (28.2–42.5)	37.8 (30.8–45.4)	855	20.8 (15.4–27.4)	23.2 (17.6–29.9)
Asian/Pacific Islander	204	33.8 (19.4–52.1)^¶^	33.8 (21.3–49.0)^¶^	207	N/A**^††^**	19.4 (11.2–31.4)^¶^
American Indian/Alaskan Native	190	51.2 (36.3–65.8)	51.2 (38.5–63.8)	187	26.2 (16.2–39.4)^¶^	33.0 (22.7–45.2)^¶^
Other non-Hispanic race or multiracial	368	33.8 (23.2–46.5)	41.2 (30.5–52.7)	379	17.9 (11.2–27.3)^¶^	24.1 (15.9–34.8)^¶^
**Education level**						
Less than high school diploma	443	43.1 (33.1–53.7)	44.1 (35.7–52.8)	444	33.6 (24.2–44.6)	31.3 (23.8–39.9)
High school diploma	2,404	37.0 (32.2–42.1)	37.7 (33.3–42.3)	2,443	21.2 (16.9–26.3)	21.4 (17.6–25.8)
College or tech school	6,765	35.1 (32.1–38.3)	35.9 (33.2–38.6)	6,960	13.5 (11.2–16.1)	14.1 (12.2–16.3)
Disability status**						
Yes	1,904	45.4 (39.1–51.9)	46.9 (40.2–53.7)	1,930	30.3 (24.1–37.4)	30.1 (23.8–37.2)
No	7,695	34.4 (31.8–37.2)	35.5 (33.0–38.1)	7,901	14.7 (12.6–17.1)	15.7 (13.6–18.0)

**Abbreviations:** CI = confidence interval; RSE = relative standard error.

* Estimates are age-standardized to the 2000 projected population for the United States.

**^†^** Binge drinkers were defined as respondents who consumed ≥4 drinks per occasion during the preceding 30 days for women and ≥5 drinks for men. An occasion is generally defined as 2–3 hours.

^§^ Respondents were from 17 states (Connecticut, Florida, Hawaii, Indiana, Kansas, Kentucky, Massachusetts, Michigan, Minnesota, Montana, Nebraska, New Mexico, New York, Oregon, Texas, Washington, and Wisconsin) and the District of Columbia. Florida and Massachusetts only obtained landline data.

^¶^ RSE = 0.20–0.30.

** Respondents were asked, “Are you limited in any way in any activities because of physical, mental, or emotional problems?” and “Do you now have any health problem that requires you to use special equipment, such as a cane, a wheelchair, a special bed, or a special telephone?” Persons who responded yes to either question were classified as having a disability.

**^††^** Estimate not available (N/A) if the RSE >0.30.

**TABLE 3:** Age-standardized* prevalence estimates of adult binge drinkers**^†^** who reported being asked an alcohol use screening–related question and advised to reduce their level of drinking by a health care provider at last routine checkup in the past 2 years, by state — Behavioral Risk Factor Surveillance System, 17 states and District of Columbia, 2014.

State/District	Sample size	Prevalence % (95% CI)
District of Columbia	333	31.0 (24.8–38.0)
Hawaii	623	28.2 (23.5–33.4)
New Mexico	495	23.5 (18.9–28.8)
Florida^§^	167	22.8 (14.8–33.5)
Texas	904	22.0 (18.0–26.5)
Indiana	310	18.8 (14.0–24.9)
Washington	762	18.7 (15.4–22.7)
Connecticut	560	18.6 (14.6–23.4)
Kentucky	451	18.2 (13.8–23.6)
Massachusetts^§^	214	18.2 (11.5–27.7)
Montana	541	15.6 (12.0–20.0)
Oregon	334	15.4 (11.5–20.2)
Michigan^§^	266	14.9 (9.7–22.2)
New York	230	14.2 (9.6–20.4)
Nebraska	875	14.0 (10.6–18.3)
Wisconsin	775	13.3 (10.5–16.9)
Kansas	377	12.7 (9.2–17.3)
Minnesota	1,638	12.0 (10.2–14.1)

**Abbreviation:** CI = confidence interval.

* Estimates are age-standardized to the 2000 projected population for the United States.

^†^ Binge drinkers were defined as respondents who consumed ≥4 drinks per occasion during the preceding 30 days for women and ≥5 drinks for men. An occasion is generally defined as 2–3 hours.

^§^ Estimate is unreliable because relative standard error = 0.20–0.30.

## Discussion

In 2014, only one in three binge drinkers was asked about alcohol use and advised about risky or harmful drinking levels. Further, only one in six binge drinkers was asked about alcohol use and advised by a health professional to reduce their drinking. A previous CDC report of 2011 BRFSS data found that only one in six U.S. adults reported ever talking with a health professional about alcohol. Because of differences in the methodologies between this prior study and the current study, including the specific ASBI questions asked, populations assessed, and timeframes of reference for the interaction with the health professional (lifetime or ever versus the last 2 years) (*4*), the findings are not directly comparable; however, both reports indicate that critical aspects of ASBI are not occurring routinely. Further, it might be that health professionals are asking about alcohol use on a form and not actually talking with their patients about their consumption. A conversation between patient and provider is traditionally a component of ASBI. While most adults reported being asked about alcohol use during a checkup, only one in three reported being asked about binge-level consumption, even though screening for binge-level consumption is recommended. Without proper screening^¶¶^ and assessment, health professionals will not know which patients could benefit from a brief intervention, treatment (which might include pharmacotherapy), or a referral to treatment for alcohol dependence. A recent estimate of the prevalence of past-year alcohol dependence was 3.5% of the total U.S. adult population. Only 10.2% of all excessive drinkers were considered to have past-year alcohol dependence (*5*).

Among binge drinkers who were asked about their alcohol use, males and persons with disabilities were more often advised about harmful levels of alcohol use and advised to reduce intake than were females and persons without disabilities. Persons with disabilities might have frequent interactions with the health care system, be older, in poorer physical and mental health, or have co-morbidities that increase their chances of being counseled on alcohol use (*6*). State variations in ASBI provision could be related to differences in levels of consumption and alcohol-related health problems, insurance coverage, or other factors influencing the behavior of health care providers, such as the socioeconomic status of their patients.

Despite current policies that support the provision of ASBI, including recommendations for its use by the USPSTF and the related Affordable Care Act requirement that many health plans cover it,*** and availability of evidenced-based clinical and implementation guidelines, these data indicate that all elements of ASBI are not routinely implemented in clinical settings, especially screening as recommended and brief intervention for persons who are screened and found to drink excessively. Federal agencies have supported initiatives to increase delivery of ASBI. For example, since 2014, CDC has funded FASD Practice and Implementation Centers^†††^ and national partners^§§§^ to focus on systems-level practice change to make ASBI standard in primary care, and published an implementation guide in 2014 for primary care medical practice settings.^¶¶¶^ The Substance Abuse and Mental Health Services Administration (SAMHSA) has funded state and medical education cooperative agreements and grants for ASBI since 2003. SAMHSA also has a national hotline that provides referrals to local treatment facilities, support groups, and community-based organizations (1–800–662-HELP [4357]; online treatment locators) and provides information about billing codes for ASBI reimbursement. The National Institute on Alcohol Abuse and Alcoholism has published clinical guidelines for conducting ASBI.**** In addition, The Community Guide evaluated the effectiveness of electronic screening and brief intervention for excessive alcohol use (which involves the use of computers, telephones, and social media) and recommended it in 2012.^††††^

The findings in this study are subject to at least four limitations. First, the data are self-reported, which can lead to social desirability and reporting biases. Second, because the data were obtained from 17 states and DC, prevalence estimates might not be nationally representative. Third, BRFSS does not collect information from persons living in some institutional settings (e.g., prison), and the prevalence of ASBI might differ in these groups. Finally, the survey median response rate was 42.7%, raising the possibility of response bias.

ASBI is effective in reducing excessive alcohol use, and if used routinely in primary care, could have a significant population-level benefit, particularly if other effective community-level strategies (e.g., increasing alcohol taxes and regulating alcohol outlet density) (*2*) are also implemented. Systems-level changes, such as including ASBI in electronic health records with appropriate prompts and screening tools, might facilitate implementation (*7*). Including ASBI measures in performance measurement programs, such as the Healthcare Effectiveness Data and Information Set, might also promote implementation (*8*). Further, the provision of ASBI by physicians and nonphysicians, including nurses, health educators, or other health professionals, has been shown to increase implementation and decrease consumption if multiple implementation strategies are used (i.e. patient, professional, and organizational approaches) (*9*). Kaiser Permanente of Northern California serves 3.8 million members in 15 counties and implemented ASBI in 54 adult primary care clinics in 11 medical centers as a part of the Alcohol Drinking As a Vital Sign (ADVISe) study (*10*). Additional systems-level implementation of ASBI, consistent with recommendations and with the provision of evidence-based community-level strategies, holds promise for broad level reduction of excessive alcohol use.

**Summary**

**What is already known about this topic?**

Although excessive or risky alcohol use is a major preventable cause of morbidity and mortality, according to 2011 CDC data, only one in six U.S. adults reports ever having a conversation with a health professional about alcohol use. It has been recommended by the U.S. Preventive Services Task Force (USPSTF) that all U.S. adults aged ≥18 years be screened for alcohol misuse and receive brief counseling if needed.

**What is added by this report?**

Findings from a 5-question module on alcohol screening and brief intervention (ASBI) using Behavioral Risk Factor Surveillance System survey data from 17 states and the District of Columbia in 2014 indicate that only one in three binge drinkers was asked about alcohol use (in person or on a form) and advised about risky drinking levels. Further, only one in six binge drinkers was asked about alcohol use (in person or on a form) and advised to reduce their drinking by a health professional.

**What are the implications for public health practice?**

Continued work at the health systems and individual practice levels is needed to implement ASBI per the USPSTF recommendation. If ASBI was provided as recommended in all appropriate medical settings, and coupled with recommended, evidence-based community interventions, preventable morbidity and mortality associated with excessive alcohol use might be reduced.

## Footnotes

* Excessive drinking is defined as binge drinking (≥4 drinks for women, ≥5 drinks for men on an occasion), high weekly consumption (≥8 drinks for women, ≥15 drinks for men in a week), and any drinking by pregnant women or persons aged <21 years. https://www.cdc.gov/alcohol/fact-sheets/alcohol-use.htm. Risky drinking includes exceeding daily and/or per occasion limits (≥4 drinks for women, ≥5 drinks for men on an occasion or in a day) and/or exceeding weekly drinking limits. Further, pregnant women and persons aged <21 years are recommended to not drink at all, and for them, any use is considered risky. Persons prescribed certain medications, or with some medical diagnoses, or engaging in some activities that might be negatively affected by alcohol use might need to drink less and communicate with their health professional about drinking in relation to their health. https://www.cdc.gov/ncbddd/fasd/documents/alcoholsbiimplementationguide.pdf;

† World Health Organization. Global Status Report on Alcohol and Health 2014. https://www.who.int/substance_abuse/publications/global_alcohol_report/en/.

§ Per the 2013 USPSTF recommendation for alcohol misuse screening and counseling. https://www.uspreventiveservicestaskforce.org/Page/Document/RecommendationStatementFinal/alcohol-misuse-screening-and-behavioral-counseling-interventions-in-primary-care. The USPSTF considers three tools as instruments of choice for screening for alcohol misuse in the primary care setting: Alcohol Use Disorders Identification Test (AUDIT) (http://apps.who.int/iris/bitstream/10665/67205/1/WHO_MSD_MSB_01.6a.pdf), AUDIT-Consumption (AUDIT-C), and single-question screener (for example, the National Institute on Alcohol Abuse and Alcoholism (NIAAA) recommends asking, “How many times in the past year have you had 5 [for men] or 4 [for women and all adults aged ≥65 years] or more drinks in a day?)” These measures include binge-level alcohol consumption occurring on an occasion or in a day.

¶ Connecticut, Florida, Hawaii, Indiana, Kansas, Kentucky, Massachusetts, Michigan, Minnesota, Montana, Nebraska, New Mexico, New York, Oregon, Texas, Washington, and Wisconsin.

** The module lead-in question was “Healthcare providers may ask during routine checkups about behaviors like alcohol use, whether you drink or not. We want to know about their questions.”

†† The American Association of Public Opinion Research Cooperation Rate is the number of complete and partial interviews divided by the number of contacted and eligible respondents. https://www.cdc.gov/brfss/annual_data/2014/pdf/2014_DQR.pdf.

§§ A Response Rate is an outcome rate with the number of complete and partial interviews in the numerator and an estimate of the number of eligible units in the sample in the denominator. https://www.cdc.gov/brfss/annual_data/2014/pdf/2014_DQR.pdf.

¶¶ CDC recommends the use of the AUDIT (US) version for screening/assessment and the Alcohol Use Disorders Identification Test 1-3 (US) for screening as well as the NIAAA single-question screener as per https://www.cdc.gov/ncbddd/fasd/documents/alcoholsbiimplementationguide.pdf.

*** The Patient Protection and Affordable Care Act of 2010 requires that nongrandfathered private health plans provide coverage without cost-sharing for services that have in effect an “A” or “B” recommendation from the USPSTF. Because the USPSTF issued a “B” recommendation for alcohol misuse screening and behavioral counseling interventions in adults aged ≥18 years, this must be covered by such plans, Section 1001 of the Patient Protection and Affordable Care Act, Public Law 111-148, 2010. https://www.gpo.gov/fdsys/pkg/PLAW-111publ148/html/PLAW-111publ148.htm. https://www.uspreventiveservicestaskforce.org/Page/Name/uspstf-a-and-b-recommendations.

††† CDC Fetal Alcohol Spectrum Disorders (FASD) Practice and Implementation Centers or PICS are Baylor College of Medicine in collaboration with the American Academy of Family Physicians, the University of Alaska Anchorage in collaboration with the American College of Nurse-Midwives and the National Association of Nurse Practitioners in Women’s Health, the University of California, San Diego in collaboration with the Association of Women’s Health, Obstetric and Neonatal Nurses, the University of Missouri, the University of Nevada, Reno in collaboration with the American Association of Medical Assistants, and the University of Wisconsin. https://www.cdc.gov/ncbddd/fasd/training.html.

§§§ CDC FASD National Partners are American Academy of Pediatrics, American College of Obstetricians and Gynecologists, University of Pittsburgh School of Nursing, University of Texas at Austin School of Social Work, National Organization on Fetal Alcohol Syndrome. https://www.cdc.gov/ncbddd/fasd/training.html.

¶¶¶ https://www.cdc.gov/ncbddd/fasd/documents/alcoholsbiimplementationguide.pdf.

**** https://pubs.niaaa.nih.gov/publications/Practitioner/CliniciansGuide2005/guide.pdf.

†††† https://www.thecommunityguide.org/topic/excessive-alcohol-consumption.
